# Adaptive, reversible, hypothalamic reproductive suppression: More than functional hypothalamic amenorrhea

**DOI:** 10.3389/fendo.2022.893889

**Published:** 2022-10-19

**Authors:** Jerilynn C. Prior

**Affiliations:** ^1^ Endocrinology and Metabolism, Centre for Menstrual Cycle and Ovulation Research, Division of Endocrinology, Department of Medicine, University of British Columbia, Vancouver, BC, Canada; ^2^ School of Population and Public Health, University of British Columbia, Vancouver, British Columbia, Canada; ^3^ BC Women's Health Research Institute, Vancouver, British Columbia, Canada

**Keywords:** hypothalamic oligo-amenorrhea, subclinical ovulatory disturbances, hypogonadotropic hypogonadism, cyclic progesterone therapy, menopausal hormone therapy, combined hormonal contraceptive therapy, short/insufficient luteal phase, anovulation

## Introduction

“Functional hypothalamic amenorrhea” (no flow for 3–6 months in premenopausal women with low gonadotrophin levels) is the focus of much clinical attention (1) as well as fundamental scientific research (2). For this special issue, amenorrhea is framed as “estrogen deficiency”.

What if amenorrhea were the “tip of the iceberg” of several differently presenting clinical entities that could all be considered “reproductive suppression” and in which there was no evidence that each, itself, was a disease? Suppose that all of these reproductive-suppression disturbances shared the characteristic of being preceded by some sort of “threat” or “stressor” that could be related to undernutrition/weight loss, illness, or social or emotional issues? If four different reproductive entities (amenorrhea, oligomenorrhea, normal cycle anovulation, and normal cycle short luteal phase) involved a lack of normal flow and/or missing of normal ovulation but also had normal or low gonadotrophin levels, could they not be part of a common process? What about it they were all usually spontaneous reversible with or without treatment? Would that not further support the hypothesis that they are part of one fundamental reproductive-suppression process? What if there was evidence that all four of these could be considered adaptive or a way of protecting the health and wellbeing of the woman experiencing them and preventing pregnancy when that woman was under duress? These are important questions.

The purposes of this “opinion piece” are, first, to describe the clinical evidence that “functional hypothalamic amenorrhea” shares many characteristics with oligo-menorrhea but that normal cycle intervals lacking ovulation and normal-length cycles with short or insufficient luteal phases are quite different hormonally and in their “visibility”. Second, this perspective will describe the contexts of women’s lives in which adaptive, reversible hypothalamic reproductive-suppression changes occur. This will emphasize how similar the responses of the reproductive system are to nutritional, psychosocial/emotional, severe illness, or exercise overtraining “stressors” that can be viewed as allostatic responses to “threats”. The third section will review the gradations of ovarian hormonal production characteristic of these four clinical reproductive entities and the likely neuroendocrine pathways leading to them. Finally, this review will present evidence that collaborative learning with the involved woman to understand and reverse the originating physiological or emotional stressors plus cyclic oral micronized progesterone (cyclic progesterone therapy) is a more physiological and likely effective therapy than the current treatment with “the pill” (combined hormonal contraception (CHC)) or menopausal hormone therapy (MHT). This new therapy is already evidence-based and feasible but requires further scientific study and knowledge translation to health professionals and women.

## A spectrum of clinical entities of hypothalamic reproductive suppression

**Figure 1:**
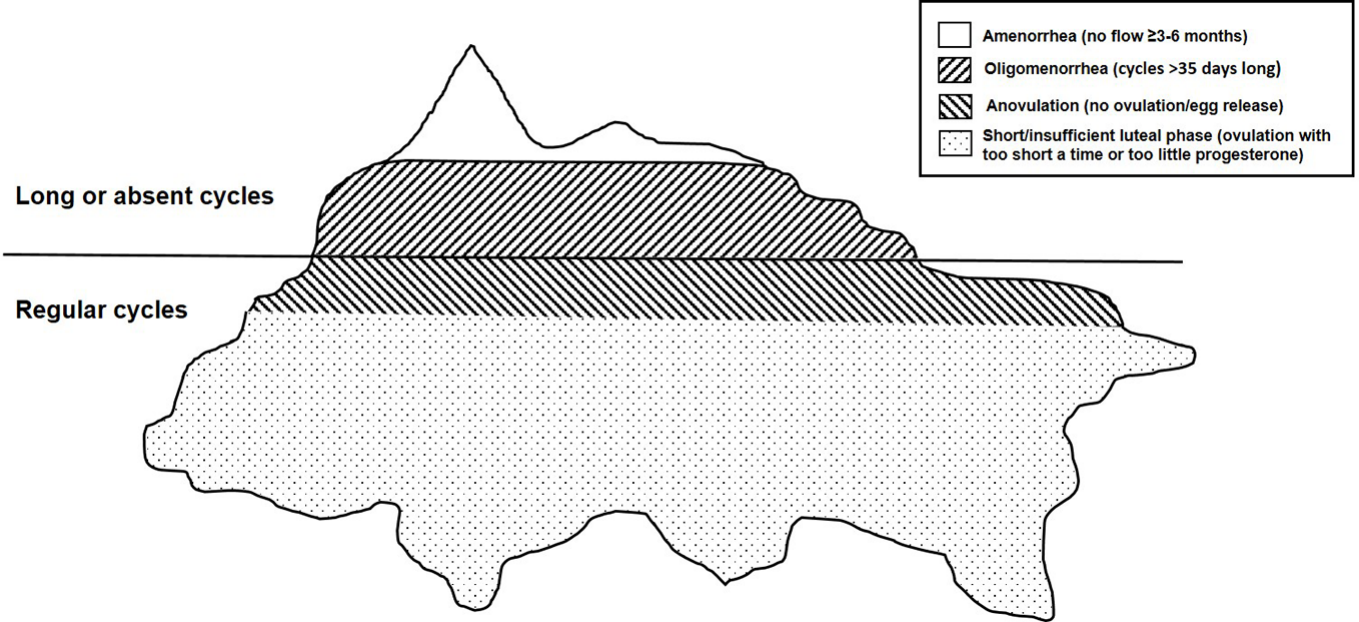
This drawing depicts an iceberg floating in the ocean as an analogy for the four adaptive, reversible, hypothalamic reproductive-suppression entities: amenorrhea, oligomenorrhea, and clinically normal cycles with anovulation and short luteal phases (the latter two called subclinical ovulatory disturbances (SOD)).

There are four clinically distinct entities that together make up hypothalamic reproductive suppression. **Figure 1** shows a typical iceberg as an analogy for the four reproductive-suppression entities. Very little of its total bulk is visible above the waterline; being visible means having obvious disturbances in the time between episodes of flow or menstrual cycle lengths. Amenorrhea is the very tip, with oligomenorrhea also making up the visible rest of this huge hunk of floating ice. The largest portion of this “iceberg” analogy of hypothalamic reproductive-suppression entities is what are called “subclinical ovulatory disturbances” (SOD) because they occur within normal-length cycles and are thus virtually invisible yet involve changes related to ovulation and progesterone. This is an apt analogy because both anovulation (which accurately means lack of egg release), and short luteal phases, provide insufficient progesterone production to preserve pregnancy, (because of too short a luteal phase length or lower than optimal progesterone levels) are currently controversial; often even the existence of SOD is denied (3).

As in the iceberg analogy, hypothalamic reproductive suppression also appears to occur as a *graded response* with the most physiologically disruptive being the least common (amenorrhea) and those that are least “deficient” being very prevalent but “invisible”. These reproductive-suppression changes are termed “adaptive” because they all teleologically function to prevent pregnancy. They also appear to prioritize the preservation of estradiol over progesterone levels. In terms of severity of the physiological disruption, amenorrhea is first followed by oligomenorrhea, then anovulation in clinically normal cycles, and finally normal-length cycles with shortened luteal phases. These latter two suppressive entities preserve normal/near-normal estradiol levels and are thus the least divergent from optimal physiology. We will discuss hormonal levels in each of these aspects of hypothalamic reproductive suppression in a later section.

Cycle length (e.g., amenorrhea) and ovulatory disturbances (e.g., SOD), which fall within the “hypothalamic” category, are not associated with “organic pathology”, overt genetic conditions, or known diseases (1). Therefore, to make the diagnosis of any of these four entities, it is necessary to exclude conditions such as pregnancy, prolactin-producing or other pituitary tumors, primary ovarian insufficiency or early perimenopause (4), severe systemic illnesses, and any hypergonadotropic diseases (1). “Polycystic ovary syndrome” (PCOS) is a prevalent reproductive imbalance that must also be excluded (5). It is mentioned separately because of its 10% prevalence in menstruating women in population-based data from multiple countries and of many races/ethnicities (6). PCOS shares with oligomenorrhea, hypothalamic reproductive suppression and ovulatory disturbances with SOD but is different because it is classically characterized by higher-than-physiological androgen levels or clinical hyper-androgenism (5); androgen levels tend to be normal or low in adaptive hypothalamic reproductive-suppression entities.

We start with amenorrhea in defining and describing and providing the prevalence of each of the four reproductive-suppression entities in turn. From a population perspective, pregnancy is the most common reason for the lack of menstruation for more than 3 months. Amenorrhea, with no flow for 3 or more months, occurs most commonly in the adolescent years (7). Secondary amenorrhea occurs after the establishment of cycles following menarche; it was present in 4.6% of women aged 15–44 years in a 1-year retrospective population-based Danish questionnaire study of ~4,000 women (response rate 78%) (7). However, in a population-based observational 1-year study in 2,400 adolescent women (mean age 15 years) from the Netherlands, only 0.5% had amenorrhea: half of these were primary amenorrhea (menarche had not yet occurred) and half were secondary (8).

The second reproductive suppression involves a longer-than-usual cycle (>35 days), called “oligomenorrhea” in classical terminology. Like amenorrhea, it is also usually obvious (above the waterline in the iceberg analogy) and thus may come to medical attention. The Danish study on menstruating women of all ages showed a 1% prevalence of oligomenorrhea (9). The Netherlands’ study on adolescents showed oligomenorrhea had a 5.5% prevalence (8). In adult, non-menopausal, Canadian women aged 25–45 years in a population-based cohort, 10.1% reported having *ever* experienced oligomenorrhea during their lifetimes (10).

Ovulatory disturbances are more prevalent than amenorrhea or oligomenorrhea but less well studied, especially in population-based samples. A single-cycle “point prevalence” investigation of ovulation in a population-based study in over 3,000 Norwegian women aged 20–49 years with normal cycle lengths of 21–35 days long found that SOD occurred in 24%–37% of cycles (11). If a woman had recorded the date of her next flow (as planned, but that actually happened for fewer participants), the prevalence of ovulatory disturbances was still almost a quarter of all cycles (11). No study has since attempted to replicate this study design.

The prospective incidence of subclinical ovulatory disturbances, in random populations of premenopausal women, is currently unknown. SOD occurred in 44% of all normal-length cycles in a prospective convenience sample of 123 young adult Metro Vancouver women aged 19–35 years who were commonly post-secondary students of mean age 22 who were studied over 2 years (12). During this 2-year study, very few experienced amenorrhea (0.9%), and 12% experienced oligomenorrhea (12). Also, in 154 women aged 18–21 years, living in a dormitory and studying over the 3 years of their nursing program at a military institution in South Korea, 69.9% had normal cycle intervals but were non-ovulatory during the school months (13). Finally, in prospective data over 1 year in 53 healthy, normal-weight, initially normally cycling and ovulating women aged 20–41 who kept continuous cycle/ovulation records, short luteal phases occurred for 25% and anovulation for 6.2%; all cycles, however, were of 21–35 days, which were normal (14).

## Origins of etiologies of adaptive, reversible, hypothalamic reproductive suppression

All of these four types of reproductive-suppression experiences are related to situational/environmental stressors, are generally reversible, and are not a manifestation of, or in themselves, a disease (15). A relatively recent history of medical approaches to women’s long-distance running will provide some insight into adaptive reproductive suppression. As an Assistant Professor of Endocrinology in the early 1980s and trying to find my niche in the academic world, women were officially prevented from running distances longer than 5 km. However, women insisted, and I was happy to see athletic women successfully running increasingly long distances. However, almost immediately, there were multiple reports of amenorrhea in runners; the most scientific study was by Harvard’s Dr. Janet W. McArthur, a highly respected women’s reproduction researcher, who described amenorrhea in normal-weight athletic women (16). By 1997, there was an official American College of Sports Medicine entity called the “The Female Athlete Triad”, which included amenorrhea, eating disorders, and low bone mineral density (BMD) (17).

In 1984, McArthur and colleagues published an exercise-training study of long-distance running in university-student women to examine the effects of weight loss as well as the training on women’s cycles and hormonal characteristics (18). A prospective two-cycle study was performed on 28 “untrained college women” with a mean gynecological age of 10 years. They were randomly assigned to weight loss and weight stable groups and lived in a dormitory in a different city during the study. Results showed that in these two groups—all of whom were running 4 miles/day by week 1 and 10 miles per day by week 5—only one woman (8%) in the weight maintenance group but 75% of those in the weight loss group had oligomenorrhea. When examining hormonal characteristics based on daily urine hormonal excretions, luteal phase shortening or anovulation occurred at a similar rate in both groups (66 and 60%, NS). Loss of the luteinizing hormone (LH) peak was twice as common in the weight loss than in weight maintenance groups (44% versus 81%, *p* = 0.05) (18). None of the women developed amenorrhea, and all of the cycle or ovulation changes had reversed to normal within 6 months (18). It is possible that some of the reproductive changes were due to dormitory housing (living closely with strangers) as well as weight loss, not just the running exercise. Nevertheless, the concept of “athletic amenorrhea”—based on cross-sectional studies, not considering reproductive maturation, exercise adaptation, nutritional/caloric imbalances, or the social situation—persisted.

By 1990, I had performed and published a prospective, observational 1-year study on 66 normal-weight, non-smoking community-dwelling women aged 20–41 years. I documented that the marathon-training and recreational female runners within a mixed exercise pattern cohort had similar 1-year menstrual cycle lengths, ovulatory characteristics, and BMD changes as those doing no regular exercise (19). It is likely that study eligibility criteria—two consecutive normal-length, normally ovulatory cycles—explained the lack of incident oligo/amenorrhea. However, about a quarter of cycles had short luteal phases (<10 days (20) by quantitative basal temperature (QBT)), and 4% showed anovulation. In a total of almost 750 menstrual cycles, incident ovulatory disturbances were experienced at least once during the year by 80% (53 of 66) of the participants (19). In this prospective, observational study, the lack of amenorrhea during training for long-distance running was subsequently repeated by two other community-based studies (21, 22). “Athletic amenorrhea”, however, is still commonly believed to be an entity in 2022.

In a follow-up of this cohort 5 years later, we also learned that “cognitive dietary restraint” (meaning the stressful worry that eating certain foods might lead to weight gain) was associated with ovulatory disturbances and potentially with BMD loss (23) despite woman participants maintaining normal and stable body weights. Cognitive dietary restraint has since repeatedly been associated with increased cortisol excretions (12, 24), indicating that this attitude toward food is related to stress.

I perceived that the “female athlete triad” was based on the erroneous belief that women’s intense physical activity was not socially acceptable and thus would cause pathology. By contrast, scientific evidence that I and others had collected indicated that the short luteal phases and anovulatory changes within regular cycles are not diseases but adaptive responses to the increasing energetic load and other physiological changes that occurred with increasing exercise (25).

In the 1980s, I postulated that the hypothalamic–pituitary–ovarian axis could become “conditioned” to increasing exercise; this conditioning was similar to documented exercise-related changes in the muscular, cardiovascular, and respiratory systems (26). This postulation of an *adaptive system* was further confirmed by data showing that ovulatory disturbances and infertility occurring during marathon training can be reversed to normal with decreasing physical activity (27). Reversibility had been confirmed earlier (18) and was supported by data showing that menopausal women admitted to the hospital with life-threatening illnesses such as pneumonia or heart failure had premenopausal levels of LH; with recovery and before hospital discharge, LH levels returned to their normal menopausal high levels (28).

By 2014, the pathology-based concepts of women’s exercise causing amenorrhea, anorexia, and osteoporosis had been (hopefully) replaced by the International Olympic Committee consensus position on Relative Energy Deficiency—Sport (RED-S) (29). “Relative energy deficiency” meant that the increased energetic requirements of exercise were not being met even if weight remained stable. RED-S is applicable to athletic men as well as women and associated with a myriad of other adaptive changes in nutrition and bone in addition to reproduction (29). RED-S documented the physiological responses of the immunological, metabolic, psychological, muscle/non-bone connective tissue, hematological, cardiovascular, and even gastrointestinal systems (29). However, RED-S, as presently constructed, does describe subclinical ovulatory disturbances as a fine-tuned understanding of the spectrum of women’s adaptive reproductive changes.

Despite the prevalence of SOD, in 2022, the general concept remains: *a regular, normal-length menstrual cycle is always normally ovulatory* (3). We can now ask: Does the concept of adaptive reproductive responses fit with *experimental evidence* in female monkeys or women? For illustrative purposes, only two of the many studies will be briefly described. An experimental study showed the shortening of the luteal phase as well as oligomenorrhea in some cynomolgus monkeys exposed to increasing and multi-dimensional stressors (30). Recent data also showed that 125 otherwise healthy community-dwelling premenopausal women exposed to the many changes necessitated by the COVID-19 pandemic experienced a high prevalence of anovulatory and short luteal phase cycles, although cycle lengths remained basically normal (31).

In the monkey experimental trial, the investigators randomized mature female cynomolgus monkeys into three groups: moving cages (next to strange monkeys, a mild psychosocial stressor for monkeys), increasing exercise with decreased dietary intake causing weight loss, and increasing exercise/decreased food plus moving cages (30). Results showed that these monkeys were resilient and without cycle length or ovulation changes in response to the first two experimental manipulations. However, the third group subjected to both psychosocial (moving cages) and exercise/energy insufficiency stresses showed both shortening of the luteal phase lengths and lengthening of reproductive cycles (30).

An “epidemic” of SOD in women in a single-cycle, observational “experiment of nature” occurred in Metro Vancouver during the SARS-CoV-2 pandemic (31). This study recruited 125 women during the first 1.5 years of the COVID-19 crisis (31). SOD occurred in 68 of 108 community-dwelling premenopausal women with ovulation-evaluable data; 13 women discontinued the trial, and four women’s temperature records were uninterpretable by the QBT ovulation documentation method. The vast majority (91%) of these milder hypothalamic reproductive suppressions of ovulation occurred within clinically normal cycles that are 21–35 days apart (31). This prevalence of oligomenorrhea was not different from that observed in a similar design study in the same community we had conducted over a decade earlier (31).

In this study, the “stressor” was the COVID-19 pandemic’s “lockdowns” with changed work (32), shopping, and childcare patterns, plus the threat of illness for one’s self or loved ones, loss of usual social activities, and increased sexual violence (33). In this Menstruation and Ovulation Study 2, women’s daily Menstrual Cycle Diary^©^ (34) data recorded their menstrual cycle-related and other experiences. Women with ovulatory disturbances (63%) documented significantly higher feelings of frustration, anxiety, depression, and “outside stresses” than did the 37% of women whose cycles were normally ovulatory (31).

In summary, largely invisible, regular, normal-length cycles with the ovulatory disturbances of anovulation and short luteal phases, like the more obvious and intense reproductive suppressions of amenorrhea and oligomenorrhea, are part of a spectrum of allostatic, adaptive responses (26, 35). These changes are documented to *reverse to normal* when stressors are decreased (2, 13, 27). Interestingly, as our cyclic progestin therapy research showed (discussed in the therapy section) (36), during reversal and recovery, there is a stepwise change to the next least disruptive reproductive-suppression entity. Berga and Yen illustrated this in their review of amenorrhea (2), figure 18–25. Energy balance is a fundamental requirement for normal reproduction, because, directly or indirectly, balanced energy intake and expenditure are commonly disturbed when reproduction is suppressed. In addition, high luteal phase progesterone levels cause an approximately 300-kilocalorie increased energy requirement (37). Therefore, it makes sense to decrease progesterone levels, as occurs in SOD, and thus decrease the risk of energy imbalance. By the time estradiol (E2) levels have become low in amenorrhea, or lower-than-normal in oligomenorrhea, ovulation is already decreased and progesterone (P4) levels are already low (19), as we will discuss in the third section of this review.

## The endocrinology of adaptive, reversible, hypothalamic reproductive suppression

Women’s health concepts today primarily focus on estradiol (E2) and its admittedly strong and important growth-stimulating actions in every cell in the body (38). Estradiol, however, is only one of two major ovarian hormones (E2 and progesterone [P4]). It was documented in 1990 that E2 and progesterone (P4), in multiple tissues and many species, have important but differing and counterbalancing cellular effects (38). The evidence that both estradiol and progesterone are important (39) has largely been ignored. Estradiol’s central, and often solo, place in women’s health is so often repeated as to currently have become a *cultural* concept (40).

**Figure 2:**
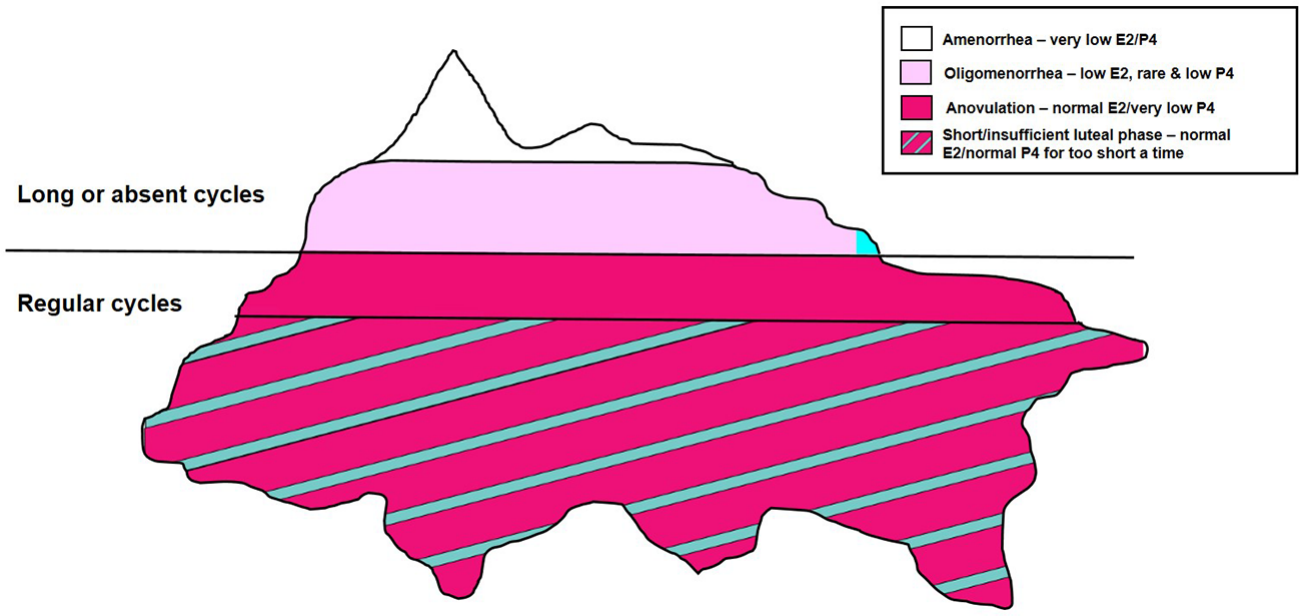
This identical drawing to **Figure 1** shows the mean levels of estradiol (E2) and progesterone (P4) in each of the four clinical entities of hypothalamic reproductive suppression.

**Figure 2**, which is identical to **Figure 1** except for its color coding, utilizes colors and their shades to show usual ovarian hormonal levels: deep pink = estradiol (E2) and turquoise = progesterone (P4). The suite of hypothalamic reproductive-suppression entities (amenorrhea, oligomenorrhea, and normal-length cycles with anovulation and short luteal phases) is now illustrated by their hormonal characteristics. Normal-length, normally ovulatory menstrual cycles provide references for these usual hormone levels (41–43).

Estradiol levels change widely across the typical cycle: from low during flow in the early follicular phase, E2 rises to its mid-cycle peak, which is 220% above its own follicular phase baseline before decreasing again to low follicular phase levels in the next cycle (42). The higher the integrated E2, especially higher E2 levels during the follicular phase (43), the shorter the whole cycle length. By contrast, P4 maintains a low level throughout the majority of the follicular phase but rises to a plateau that is ideally at least 10–12 days in duration (depending if its onset is considered by QBT or using the LH peak). The P4 level during the luteal phase rises to a peak that is 1,400% higher than its follicular low level and again decreases to low by the next cycle (42). Note that the typical “Google image” of E2 and P4 in the normal menstrual cycle is inaccurate (39); it is a “graph” without measurement units; it falsely implies that estradiol and progesterone are in the same units. They are not. E2 is in pmol/L, while P4 is reported in nmol/L units—nmol/L is 1,000 larger than the pmol/L (39).

In amenorrhea, both E2 and P4 levels are lower than the usual levels found during menstrual flow (**Figure 2**). In oligomenorrhea, E2 levels are lower but variable and may briefly rise to normal levels; P4 levels are usually low. Rarely in oligomenorrhea is it possible for ovulation to occur and P4 levels to increase.

Below the "waterline", in normal-length cycles (**Figure 2**) having anovulation, E2 levels are normal or near-normal. The evidence for this statement is in population-based Norwegian data that showed ovulatory disturbed cycles had levels only 20% lower than normally ovulatory cycles (230 *vs.* 290 pmol/L) (11). In addition, in the ovulatory disturbed cycles, the 95% CI for E2 had a higher upper range of 420 *vs.* 370 in normally ovulatory cycles (11). The prospective 1-year study from our laboratory of initially normally cycling/normally ovulatory women also showed that E2 levels as well as cycle lengths were not different in normally ovulatory versus ovulatory disturbed cycles (19). By definition, however, because there is no corpus luteum in anovulation, these cycles have low levels of P4. Finally, short luteal phases in normal-length cycles have near-normal estradiol levels but shorter-than-normal luteal phase lengths and thus lower integrated cycle P4 levels.

It is likely the reason that SOD are the most prevalent of all the adaptive hypothalamic reproductive-suppression entities because they maintain near-normal estradiol levels. SOD cycles however achieve some protection for women by first suppressing the potential for pregnancy (a highly demanding metabolic, social, and personal condition) and, in addition, by avoiding the metabolic/nutritional demands of progesterone (37). High luteal phase progesterone levels raise the core temperature (generally 0.2°C), which requires approximately 300 kilocalories of increased energy (37). Thus, it makes sense, related to the fundamental reproductive necessity of energy balance, to decrease P4 levels during times of stress or duress. Evidence shows that by the time E2 levels have become low (as in amenorrhea), or averaging lower-than-normal in oligomenorrhea, ovulation is already intermittent or absent, and P4 is produced for too few days or its levels are uniformly low (19).

The origins of the lower P4 and sometimes E2 levels in the four types of reproductive suppressions involve signals from the brain (especially the limbic system where emotions are interpreted), hypothalamus, and pituitary. These brain–hypothalamic–pituitary changes leading to hypothalamic reproductive suppression are complex, dynamic, and not yet fully understood (2). Why? Because most of the studies of these adaptive, reversible entities have included no measures of each woman’s psychosocial environment, her perceived stressors, or prospective documentation of energy requirements and intakes. In other words, adaptive, reversible hypothalamic reproductive suppressions have been studied as though they were diseases, not as they have been shown to, involving environmentally responsive, dynamic changes. The women studied in describing SOD have been women who presented to medicine due to recurrent pregnancy losses and/or infertility versus normal controls (44, 45) or who were exercising versus sedentary controls (46).

Despite the lack of social/emotional and physiological contexts—a huge drawback—it is useful to start with the 1970 historical description of the “luteal phase defect” by Drs. G. S. Jones and V. Madrigal-Castro in women with infertility or pregnancy loss (44). They postulated the luteal phase defect could be caused by the hypothalamus related to “psychogenic, neurogenic or iatrogenic (drug) factors. Chronic illness, metabolic disease, nutritional defects or autoimmune diseases …” (44). They defined “luteal phase defect” as a problem with the production of progesterone for a sufficient length of time and infer that E2 production was unchanged. An important later work by Michael Soules’ laboratory studied women in the same manner showing in infertile women that LH pulsatility was overly fast (versus expected) in the early follicular phase (45). We still do not know the endocrinology leading to the rapid gonadotrophin hormone-releasing hormone (GnRH) that drives LH pulsatility in women with luteal phase defects. One could postulate that the preceding cycle were anovulatory, given progesterone’s known actions to slow LH pulse rate at the normal menstrual mid-cycle (47); without a previously ovulatory cycle, LH could be too fast at the start of the cycle. Finally, in a cross-sectionally analyzed three-cycle study comparing sedentary and moderately active female runners, De Souza and colleagues reported that late in the cycle, follicle-stimulating hormone (FSH) levels were lower in exercising women with luteal phase defects (46). In the same study, exercising women with anovulation, however, had higher FSH levels late in the cycle than in normally ovulatory cycles. The authors, however, published no explanation for why luteal phase defects and anovulation, two closely related ovulatory disturbances, would have opposite late-cycle FSH levels (46).

There are clearly suppressive effects of corticotrophin-releasing hormone (CRH)/adrenocorticotropic hormone (ACTH)/adrenal axis and endogenous opioid systems on GnRH signaling. There are also reproduction-suppressive interactions with metabolic factors such as ghrelin, leptin, and neurotransmitters such as dopamine, serotonin, and norepinephrine (1, 2). Changes in GnRH *pulsatility* appear to provide the main signals leading to menstrual cycle hormonal changes; for this reason, *levels* of the pituitary gonadotrophins, LH, and FSH often remain in the normal range (as shown in the monkey stress experiment described earlier) (30).

In summary, as shown in **Figure 2**, only the relatively rare occurrences of amenorrhea and oligomenorrhea have low or decreased E2 levels; *all four* adaptive, reversible hypothalamic reproductive-suppression entities, however, are lacking sufficient P4. If physiological stressors (say, illness, decreased caloric intake, or markedly increased physical activity) are combined with any emotional/psychological stressor, their effects on menstruation and ovulation are synergistic (as previously described) (30). Estradiol levels are maintained at near-normal during SOD, but progesterone levels are absolutely (anovulation) or moderately (short luteal phase cycles) decreased (11). The problem is that these silent ovulatory disturbances within normal cycle lengths are common and not clinically obvious.

Thus, hypothalamic reproductive suppression is an adaptive, protective, and potentially totally reversible, highly coordinated brain/hypothalamic/pituitary/ovarian response to situational and environmental stressors (27). Only when these reproductive adaptations are clinically obvious (and have abnormal cycle lengths) do they also include importantly lower E2 levels. The final important clinical issues for adaptive, reversible hypothalamic reproductive suppressions relate to its treatment. Most healthcare providers are clear that amenorrhea (of any cause other than pregnancy) requires therapy. What about oligomenorrhea? Should normal-length menstrual cycles with anovulation or short luteal phases be treated? If yes, how?

## Treatment of hypothalamic reproductive suppression

The reasons for medical treatment of hypothalamic amenorrhea and oligomenorrhea are simple—restore menstrual cycles and provide sufficient estrogen. This approach ignores the fact that progesterone levels are also “deficient” (39). However, estrogen-dominant therapies such as MHT and CHC have been the standard of care for decades. CHC has been widely touted for its “non-contraceptive benefits” (48). However, there is evidence that all hormone-based therapies, alone and without recovery of energy balance, are unlikely to allow recovery to normal cycle or ovulation. For example, a retrospective clinical study of amenorrhea, its treatment, and rates of recovery published in 2002 showed that 70 of a total of 93 women were treated with MHT and 12 with CHC, and 10 refused medical treatment (49). Over a mean follow-up of 8 years, 71% recovered menstrual cycling—74% on HRT, 80% who were untreated, and only 42% on CHC (49).

The Endocrine Society’s most recent consensus statement about “functional hypothalamic amenorrhea” takes a much more physiological approach to treatment by recommending “correcting the energy imbalance …” (1). If this and other strategies were not successful, the “short-term use of transdermal E2 therapy with cyclic oral progestin” was recommended (1). Further, these guidelines specifically and wisely recommend against CHC therapy for amenorrhea, oligomenorrhea, or irregular cycles in adolescents, although that is not yet a community standard (1). That is an important instruction since CHC use was recently associated with significant negative bone mineral density change in adolescent women (50, 51). In addition, new evidence suggests that CHC use by adolescent women is associated with an increased current risk for depression (52) as well as a higher likelihood of later-life depression (53). If amenorrhea persists, Endocrine Society guidelines suggest trying cognitive behavioral therapy (CBT) (1) for which there is a supportive randomized controlled trial (54).

These therapeutic approaches to hypothalamic reproductive suppression today are markedly improved. However, they remain insufficient for three reasons: energy imbalance is often the only stressor consistently assessed and rectified; usually, neither the physician nor the woman living with reproductive suppression is clear about the proven health consequences of *lack of treatment* of these reproductive-suppression issues (after all, avoiding menstruation is a goal for many especially younger women and seen as desirable in some cultural circles); and finally, estrogen is still viewed as the focus of the issues and used as the primary therapy, not progesterone, which is the “missing or low” hormone for all of the manifestations of hypothalamic reproductive suppression.

### All physiological and psychosocial stressors first need identification and reversal

To start, we as healthcare providers must build a relationship with the woman and address all of the multi-dimensional stressors that together we learn are related to her cycle and ovulation disturbances. Most women with hypothalamic reproductive suppressions have been exposed to major or several minor stressors usually including relative energy insufficiency and many with coexisting reproductive immaturity (because they have never established regular ovulation). I learned to detect this in women who had not used progestins or CHC by observing a small (less than 3 cm) breast areolar diameter (55). As documented earlier in the monkey experiment, it is usually a combination of types of stressors that initiate and maintain adaptive, reversible hypothalamic reproductive suppression.

In my (past) clinical practice, I described reproductive suppression to each woman as the various ways a woman’s *wise* body is protecting itself when she is experiencing some kind of threat or stress. I reassure her that she does not have a disease. She can, and likely will, totally recover. Whether or not she raises the issue of fertility, I reassure her that, in the future, if she wishes, she can eventually have a child or children. I will often then share my personal experience of amenorrhea when I first moved away from home to go to university. I knew I was not pregnant, ill, or too skinny. I told no one, and my cycle recovered when I left the dormitory and academic stress while working at home during the summer.

My next step would be to gain her trust and develop a collaborative relationship. Given the various personalities and ways in which women might end up seeing me, I had to adapt my approach to each woman. During my medical and reproductive history, I would ask her questions such as the following: “Why do you think that your (periods/ovulation) are disturbed?”, “Do you have someone you can talk with about *anything* that might be bothering you?”, “How are you managing for housing and food?”, and “What do you do for fun?” My approach was to learn about her as a whole person and to help her feel sufficiently safe to share with me.

Together, then, we could work on improving the fundamental nutritional/emotional/social stressful issues. If she were upset and concerned about potential weight gain, I would explain that we were working to help her gain *muscle and bone strength*, not increase fat. I learned to recommend, especially for someone who was underweight (body mass index (BMI) <18.5) but also for others with BMI values in the low 20s, that she increase protein and “good fats” by eating a handful of almonds after dinner and before bedtime. Usually, this was acceptable and adopted despite cognitive dietary restraint. I also always suggested regular physical activity—she could do whatever kind she *likes*, that is moderate in intensity, and that she could manage to do for half an hour on most days. Ideally, that included walking outside, with a friend and in parks or by a stream, lake, or ocean. If that was not possible, walking remains healthy and stress-reducing almost anywhere with a pet and/or while practicing mindfulness meditation (56).

I also would invite her, *whether or not she was getting periods*, to keep the Menstrual Cycle Diary^©^ (Diary) (34). If she had any flow, she would start with the first day of flow but if not, with day 1 of the month. I would explain that the reasons for diary-keeping were to help her learn to identify her feelings (frustration, anxiety, depression, self-worth, energy, and outside stresses) and how they connected with other experiences. I reassured her that the Diary was hers and she did not need to share it with me; however, she could if she wished, and most did.

In addition, the diary would make it possible for her to detect early signs of increasing estrogen production such as starting to see cervical mucus. She could then rejoice with me as she noticed the mucus became more in amount and stretchy. I also described the possibility of developing front-of-the-breast tenderness and sometimes vascular-type headaches, explaining that her body was trying hard to get her cycles and ovulation going. She may therefore have a cycle or two with higher-than-normal estrogen levels; these symptoms would always improve as she continued to recover.

### Amenorrhea, oligomenorrhea, and subclinical ovulatory disturbance health consequences

It is important that both clinicians and women living with hypothalamic reproductive-suppression disturbances understand the proven, evidence-based results of untreated amenorrhea and oligomenorrhea. These are data showing infertility as well as bone loss (provided that she does not spontaneously recover, which is also a “consequence”) (49). The population-based data are clear that chronic longer-than-normal cycles (>30 days) are associated with a later-life increased risk for fragility fractures (or osteoporosis) (57); chronically irregular premenopausal cycles also have been documented to increase later-life fracture risk (58). Although cardiovascular disease (CVD) is often mentioned as a risk in women with hypothalamic causes for absent or far-apart cycles, the evidence for heart attacks (as opposed to altered lipid levels, endothelial function, or secondary CVD markers) is very sparse. Likewise, there is little epidemiological evidence for earlier dementia or increased mortality.

However, rarely mentioned in the consequences of hypothalamic reproductive suppressions are the growing data that subclinical ovulatory disturbances, because they include sufficient estradiol but not sufficient progesterone, also pose later life health risks. *Note that earlier clinician-scientists were quite definite that “luteal phase deficiency” was a cause of infertility and recurrent early pregnancy loss* (44, 45). However, since the advent of assisted reproductive technologies, there is skepticism about those ideas. Subfertility over the next 6 months, however, was recently shown to relate to having a short luteal phase (59).

Strong data are increasingly documenting that SOD is related to loss of BMD (19, 60), although no study has yet documented increased SOD-associated fractures. In addition, evidence shows that estradiol and progesterone both have beneficial effects on endothelial function (61), and in epidemiological data, lower progesterone levels, but not lower estrogen or increased androgen levels, were linked to heart attacks occurring within a few years of the start of menopause (62). Finally, science has known for decades that estrogen without sufficient progesterone (as in estrogen-alone menopausal therapy) is a risk for endometrial cancer (63). However, little attention has been paid to the same hormonal imbalance that occurs with chronic anovulation in normal-length cycles. Physiology predicts that anovulation is associated with endometrial cancer.

Breast cancer risk is similarly confusing. Although medroxyprogesterone acetate (MPA) was associated with increased breast cancer risk in the Women’s Health Initiative estrogen–progestin randomized controlled trial (RCT), it is because MPA increases breast proliferation by acting through the glucocorticoid rather than the progesterone receptor (64). Progesterone has been shown to prevent the proliferative actions of estrogen receptor alpha (65). Estradiol with *progesterone* therapy did not increase breast cancer risk in the large prospective French E3N epidemiological data; breast cancer was significantly increased in those taking estrogen alone, and more in those on estrogen–progestin treatment (66). Thus, increasing evidence suggests that recurrent/persistent and untreated SOD may also lead to later-life increased risks for breast and endometrial cancers (39).

Cyclic progesterone therapy for hypothalamic reproductive-suppression conditions

Following the analysis of data from the 1-year prospective study in initially normally ovulatory women (19), it made sense to me that amenorrhea, oligomenorrhea, and regular cycles with anovulation and short luteal phases were all part of a spectrum of hypothalamic adaptive changes. I also believed that SOD, like amenorrhea and oligomenorrhea, needed therapy because of important BMD loss. Therefore, we designed a 1-year two-by-two factorial randomized placebo-controlled trial with a following observational month/cycle. Participants of normal-weight, otherwise healthy, and physically active women (without perimenopause, pregnancy, polycystic ovary syndrome, or hyperprolactinemia) experiencing any of the four hypothalamic reproductive-suppression changes would be randomized to cyclic MPA or an additional gram of oral calcium or their placebos (36). Active treatments were 10 mg of MPA for 10 days/cycle/month and an additional 1,000 mg of calcium. There were four therapy arms: active cyclic MPA–active calcium, active cyclic MPA–placebo calcium, placebo cyclic MPA–active calcium, and placebo cyclic MPA–placebo calcium (36). BMD 1-year change, the primary outcome, was measured using dual-energy absorptiometry methods (36). Seventy-three eligible women participated; they were 21–45 years old with a mean age of 32 and a BMI of 21.7 (36). Sixty-one women completed the 1-year study with three discontinuations in each of the four therapy groups and no serious negative adverse effects. The 1-year study was followed by an observational single untreated menstrual cycle during which ovarian hormone levels were obtained (36)

Results showed a significant 2%–3% spinal BMD gain was primarily related to cyclic MPA (ANOVA F = 19.42, *p* = 0.0001) with calcium having a borderline positive relationship (*p* = 0.065) and those on double placebos experiencing significant (2%) BMD loss (36). Menstrual cycles tended to improve; half of the completing women had normal-length, normally ovulatory cycles in the final, untreated menstrual cycle, and their mean final hormone levels were within the normal ranges for a normally ovulatory menstrual cycle (19). The cycle improvements could not be statistically related to cyclic MPA or calcium therapies, changes in body weight, or changes in exercise. However, clearly, “intervention with cyclic medroxyprogesterone did not interfere with the improved reproductive function” (36).

Why did women, despite amenorrhea and lower estrogen levels, gain BMD when treated with progestin acting through the osteoblast P4 receptor? It is because women with amenorrhea had been without flow for many months and thus had lower rates of bone resorption. Progesterone (P4) only acts on bone formation, which is the slower of the two phases of bone remodeling (resorption and formation). Thus, P4 actions are difficult to “see” when bone resorption rates are high. Evidence from multiple studies suggests that P4 is women’s bone formation-stimulating hormone (19, 60, 67, 68). The ideal E2–P4 balance is within the normally ovulatory, normal-length menstrual cycle. Therefore, if bone turnover and resorption are low, as in prolonged amenorrhea, then P4 can significantly increase BMD.

The study conducted in the 1990s needs to be repeated in a larger RCT in which cyclic oral micronized progesterone (300 mg at bedtime for 14 days/cycle) versus an identical placebo is the study design. For such a study, all women should be stabilized on sufficient calcium (approximately 300 mg with each meal and at bedtime) and vitamin D (depending on sun exposure and latitude) as well as with regular social support and/or psychological counseling. In addition, the recovery of cycles needs to be studied for three observational cycles following a 1-year therapy trial. If the results of a cyclic progesterone trial were successful, then that therapy alone should be compared in the four hypothalamic reproductive entities with documentation of changes in stressors, LH pulsatility, E2, and P4 levels, and BMD change in a double-blind trial versus the current standard of care for hypothalamic amenorrhea: transdermal E2 with cyclic progestin (1).

## Summary

There is evidence that we need a new concept about women’s hypothalamic menstrual cycle (amenorrhea and oligomenorrhea) and ovulation (anovulation and short luteal phases) suppressions. These now need to be understood as a continuum, as protective for the individual and her social network, adaptive, and potentially reversible. That means our treatments also need to fit with these new concepts. Further data are needed on the use of cyclic progesterone as a therapy. Ideally, an RCT of conventional estrogen-dominant therapy (69) versus cyclic progesterone therapy is necessary and should be followed by 1-year, post-treatment observation of menstrual cycles and ovulation differences between women in the two therapy cohorts, as well as women’s evaluation of each blinded therapy. This newer understanding of the importance, prevalence, and reversibility of women’s disturbances of reproduction, especially those that, like the bulk of an iceberg, that are “invisible” below the waterline (because they occur within regular cycles), provides a positive and science-based way forward in women’s health.

## Author contributions

The author confirms being the sole contributor of this work and has approved it for publication.

## Conflict of interest

The author declares that the research was conducted in the absence of any commercial or financial relationships that could be construed as a potential conflict of interest.

## Publisher’s note

All claims expressed in this article are solely those of the authors and do not necessarily represent those of their affiliated organizations, or those of the publisher, the editors and the reviewers. Any product that may be evaluated in this article, or claim that may be made by its manufacturer, is not guaranteed or endorsed by the publisher.
